# Pristimerin inhibits glioma progression by targeting AGO2 and PTPN1 expression via miR-542-5p

**DOI:** 10.1042/BSR20182389

**Published:** 2019-05-14

**Authors:** Zaiyu Li, Cong Hu, Yu Zhen, Bo Pang, Huanfa Yi, Xianglin Chen

**Affiliations:** 1 Department of Neurosurgery, The Sixth Hospital Affiliated to Guangzhou Medical University, Qingyuan, Guangdong, 511500, China; 2 Central Laboratory of the Eastern Division, The First Hospital of Jilin University, Changchun, Jilin 130021, China; 3 Department of Dermatology, The First Hospital of Jilin University, Changchun, Jilin 130021, China; 4 Department of Cardiology, The First Hospital of Jilin University, Changchun, Jilin 130021, China

**Keywords:** AGO2, anti-cancer, glioma cells, miRNA, Pritimerin, PTPN1

## Abstract

Glioblastoma multiform is the most common and malignant primary tumor of the central nervous system in adults, the high recurrence rate and poor prognosis are critical priorities. Pristimerin is a naturally occurring quinone methide triterpenoid isolated from the Celastraceae and Hippocrateaceae families. Its anticancer effects have garnered considerable attention; nonetheless, the mechanisms of action remain unknown. To predict the hub genes of pristimerin, PharmMapper and the Coremine database were used to identify 13 potential protein targets; protein–protein interaction, for which functional enrichment analyses were performed. Compound-target, target-pathway, and compound-target-pathway networks were constructed using Cytoscape. Biological process analysis first revealed that enrichment of these target genes correlated with negative regulation of symbiont growth in the host, and regulation of chronic inflammatory response to antigenic stimulus. Survival analysis in cBioPortal showed that protein tyrosine phosphatase, non-receptor type 1 (PTPN1) and Argonaute 2 (AGO2) might be involved in the carcinogenesis, invasion, or recurrence of diffuse glioma. In addition, we observed that low-dose pristimerin inhibited the viability of glioma cells, while miR-542-5p *in vitro*; and reduced PTPN1 expression. Notably, high-dose pristimerin induced apoptosis. Furthermore, miR-542-5p silence with siRNA in glioma cells lead to the elevation in AGO2, and decreased PTPN1 level. The effect was obviously post pristimerin treatment and miR-542-5p suppression. In conclusion, pristimerin inhibited glioma progression through AGO2 and PTPN1 expression via a canonical miRNA-mediated mechanism.

## Introduction

Glioblastoma multiform (GBM) is the most common and malignant primary tumor of the central nervous system in adults [1]. According to the histopathological characteristics, the world health organization classified gliomas in terms of malignancy into four grades (I–IV), in which IV identified the most malignant forms of GBM among the pathological types of astrocytoma [2]. In recent years, besides traditional surgery combined with radiation and chemotherapy for GBM, a new type of anti-angiogenesis therapy that targeted immunotherapy is emerging. However, GBM cure rate is still unsatisfactory, the high recurrence rate and poor prognosis are critical priorities [3].

**Figure 1:**
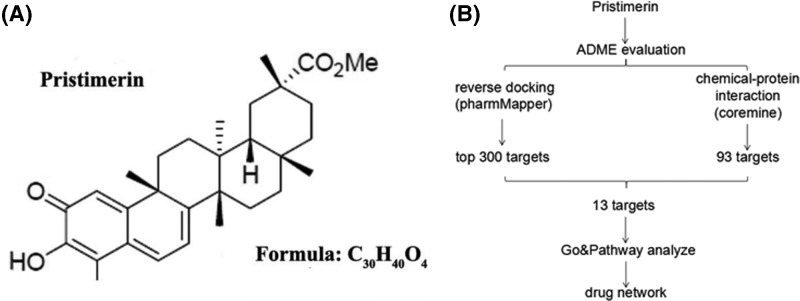
Chemical structure and pipeline for the identification of pristimerin (**A**) Chemical structure of pristimerin downloaded from the PubChem database (CID: 159516). (**B**) Pipeline for the identification of putative pristimerin targets that integrates ADME evaluation, reverse docking, chemical-protein interaction, GO, and pathway analyses, and network construction.

Pristimerin (20α-3-hydroxy-2-oxo-24-nor-friedela-1-10,3,5,7-tetraen-carboxylic acid-29-methyl ester; Figure 1A) is a naturally occurring quinone methide triterpenoid isolated from the Celastraceae and Hippocrateaceae families. Numerous natural triterpenoids structurally related to pristimerin have been discovered, including celastrol [4], ursolic acid [5], and betulinic acid [6]. Pristimerin has been reported to exert various pharmacological effects, including anticancer, anti-angiogenic, anti-inflammatory, antiprotozoal, and insecticidal roles [7]. The antitumor activities of pristimerin in a series of human cancer cell lines (e.g., colon, breast, and prostate cancers and multiple myeloma) have already been confirmed, and its antitumor function, particularly for cancer treatment and chemoprevention was associated with various molecular targets [8–13].

microRNA (miRNA) is a small non-coding RNA molecule found in plants, animals, and some viruses, which functions as RNA silencers and post-transcriptional regulators of gene expression [14,15]. microRNAs play essential roles in almost all vital life activities, such as gene transcription, post-transcriptional processing, cell differentiation, ontogenesis, heredity, and epigenetics [16]. The formation of mature miRNA involves several steps. First, RNA polymerase II promoters direct the transcription of miRNA genes into primary miRNA transcript (pri-miRNA precursor), which are sequentially processed by Drosha and Dicer nucleases III and exportin-5 to produce approx. 80nt pre-miRNA precursors and approx. 21nt double-stranded miRNA, respectively [17]. One of the double-stranded miRNAs targets the RNA-induced silencing complex (RISC) containing Argonaute (AGO) protein. AGO regulates gene expression by binding to its target gene mRNA while releasing a second chain that is subsequently degraded [18]. miRNA located in RISC bind to target genes via direct base-pairing, selectively promoting degradation or target mRNA translation inhibition, depending on the degree of complementarity [19]. The post-transcriptional gene regulation mechanism mediated by miRNA plays a vital role in a series of biological processes of tumor cells, including malignant colonization, invasion, and migration, distant metastasis, angiogenesis, and autophagy [20].

Human protein tyrosine phosphatase, non-receptor type 1 (PTPN1) gene encodes protein tyrosine phosphatase 1B (PTP1B), belongs to protein tyrosine phosphatase family and maps to 20q13.1–q13.2 [21]. PTP1B has long been known to regulate insulin and leptin receptor signaling negatively. Recently, it was reported to be aberrantly expressed in cancer cells and to function as an important oncogene [22]. Several studies have reported that PTPN1 promoted proliferation, colony formation, and migration while decreased apoptosis of cancer cell lines [22,23]. Nonetheless, the anticancer effects of pristimerin and specific mechanisms of PTPN1 in diffuse glioma deserve investigation.

In the present study, we aimed to explore the mechanism of pristimerin anticancer effect in glioma cells and attempted to figure out the potential role of biomarkers in glioma progression.

## Materials and methods

### Evaluation of drug-likeness

The TCMSP server (http://ibts.hkbu.edu.hk/LSP/tcmsp.php) is a system-level traditional Chinese medicine pharmacological database, which provides information on absorption, distribution, metabolism, and excretion (ADME). It provides an evaluation model that integrates oral bioavailability (OB), drug-likeness (DL), Caco-2 permeability, and other features [24], and helps optimizing pharmacokinetics and drug properties, such as solubility and chemical stability.

### Computational target fishing using PharmMapper and the Coremine database

PharmMapper (http://www.lilab-ecust.cn/pharmmapper/) can identify potential protein targets for small molecule compounds by pharmacophore mapping [25] and can provide the first 300–3000 targets for a given compound, sorted by appropriate scores in descending order. In comparison, the Coremine database (http://www.coremine.com/) can identify potential target proteins by chemical–protein interaction analysis of small molecules [26]; these are both powerful tools for computational target fishing. We searched for pristimerin (PubChem CID: 159526) in the PubChem database (https://pubchem.ncbi.nlm.nih.gov/). Subsequently, we downloaded a spatial data file and uploaded it to PharmMapper and Coremine databases. All parameters were set to default values, and overlapping protein targets were chosen for further investigation.

### Analysis by GeneMANIA and function enrichment analyses

GeneMANIA is a flexible, user-friendly web interface for generating hypotheses on gene function, analyzing gene lists, and prioritizing genes for functional assays [27]. The results were collated after we inputted the target genes of interest in the previous step.

GO and pathway analysis by PANTHER (http://pantherdb.org), which is a comprehensive curated database of protein families, trees, subfamilies, and functions, can be used to help understand relationships in gene expression data and provide systematic visual information on the gene of interest [28]. After the target genes of interest were uploaded following online instructions, GO, and pathway information for pristimerin was generated and enriched. To further understand the complex relationships among compounds, targets, diseases, and pathways, networks were constructed and analyzed using Cytoscape 3.3.

### Materials

Pristimerin (purity ≥ 99%, as determined using high-performance liquid chromatography), which was purchased from Paypaytech Inc. (Shenzhen, China), was prepared as a 20 mM stock solution in dimethyl sulfoxide and stored in small aliquots at −20°C.

### Cells and cell culture

Human U373 and mouse GL261 glioma cells were obtained from the Shanghai Institute of Cell Biology, Chinese Academy of Sciences (Shanghai, China). These cells were cultured in Dulbecco’s modified Eagle’s medium (DMEM) supplemented with 10% fetal bovine serum (Gibco, Grand Island, NY, U.S.A.), glutamine (2 mmol/L), penicillin (100 U/ml), and streptomycin (100 μg/ml) and were maintained at 37°C in humidified 5% CO_2_ incubators. Cells in the mid-log phase were used for further experiments.

### Cell viability assay

Glioma cells (4 × 10^4^ cells/well) were seeded onto 96-well microplate and treated with various concentrations (0, 0.25, 0.5, 1, and 2 μM) of pristimerin, separately. After 24 h, 10 μl of Cell Counting Kit-8 (CCK-8; Beyotimes, Wuhan, China) was added. After incubation for 1 h, optical density was measured using an ELISA reader (Tecan, Männedorf, Switzerland) at a wavelength of 450 nm.

### Real-time polymerase chain reaction analysis

Initially, total RNA was extracted from glioma cells using AxyPrep Multisource Total RNA Miniprep Kit (Axygen, Union City, CA, U.S.A.). The RNA quality was checked using the ratio A260/A280, and pure RNA samples were converted to cDNA using PrimeScript RT Reagent Kit with gDNA Eraser in accordance with the manufacturer’s instructions (Takara Bio, Kusatsu, Japan). The polymerase chain reaction was performed using SYBR Green based on the instruction manual (Takara Bio, Kusatsu, Japan). The primer sequences are presented in Table 1 (Comate Bioscience, Changchun, China). The relative mRNA expression of different genes was quantified using the ∆∆Ct method. U6 and β-actin were used as housekeeping genes.

**Table 1:** Oligonucleotide and primer sequence

Oligonucleotide	Primer sequence
U6	5′-CGCTTCGGCAGCACATATAC-3′ (forward),
	5′-TTCACGAATTTGCGTGTCATC-3′ (reverse)
hsa- miR542-5p	5′-CTCCTCTCGGGGATCATCAT-3′ (forward),
	5′-TATGGTTGTTCACGACTCCTTCAC-3′ (forward)
mmu-miR542-5p	5′-CTCCTCTCGGGGATCATCAT -3′ (forward),
	5′-TATGGTTGTTCACGACTCCTTCAC -3′ (forward)
β-Actin	5′-TTCAACACCCCAGCCATG-3′ (forward),
	5′-CCTCGTAGATGGGCACAGT-3′ (reverse)
PTPN1	5′-GCAGATCGACAAGTCCGGG-3′ (forward),
	5′-GCCACTCTACATGGGAAGTCAC-3′ (reverse)
AGO2	5′-TCCACCTAGACCCGACTTTGG-3′ (forward),
	5′-GTGTTCCACGATTTCCCTGTT-3′ (reverse)

### Transfection with miRNA mimics

Cells were transfected in 6-well plates with DMEM using HiPerFect transfection reagent (Qiagen, Shanghai, China) at 18 h after seeding, and miRNA inhibitor (GenePharma, Shanghai, China) were prepared at a concentration of 20 nM. Cells were harvested at 48 h after transfection.

### Apoptosis analysis by flow cytometry

Cells were treated with increasing pristimerin concentrations (0–16 μM) for 6 h, Cell apoptosis was evaluated by Annexin V-FITC/PI apoptosis detection kit (BD Biosciences) following the manufacturer’s instructions [29] and was analyzed using a FACS LSRFortessa flow cytometer.

### Statistical analysis

All data represent at least three independent experiments, and statistical analyses were performed with Prism (version 7.0, GraphPad Software, San Diego California, U.S.A.). All results are shown as mean ± SD. Statistical comparisons were performed using one-way analysis of variance. *P*-values <0.05 were considered to denote statistical significance.

## Results

### ADME-related properties of pristimerin

First, we intended to confirm pristimerin is not only a chemical compound but also a drug that can appropriate to clinical application. An in-depth study of ADME-related properties of pristimerin by TCMSP is presented in Table 2. Notably, the DL value of pristimerin was calculated to be 0.77, indicating that pristimerin may be a promising drug.

**Table 2:** Pharmacological and molecular properties of pristimerin

Name	MW	AlogP	Hdon	Hacc	OB (%)	Caco-2	BBB	DL	FASA	TPSA	RBN
PM	464.70	5.54	1	4	19.27	0.70	0.07	0.77	0.30	63.60	2

### Recognition of potential targets and analysis by GeneMANIA

To identify the potential protein targets in pristimerin, as shown in Figure 1B, the top 300 potential targets of pristimerin were predicted from all 1961 pharmacophore models obtained using PharmMapper and 93 potential targets identified using the Coremine database. Finally, to improve specificity, 13 overlapping proteins identified from both sets of results were selected for further investigation (Table 3).

**Table 3:** Putative targets of pristimerin identified by PharmMapper and Coremine

Rank	PDB ID	Gene name	Target name
1	2PQF	PTPN1	Protein tyrosine phosphatase, non-receptor type 1
2	1T2R	AGO2	Argonaute 2
3	2E19	ZEB1	Zinc finger E-box binding homeobox 1
4	1SP3	CYCS	Cytochrome *c*, somatic
5	3F3P	NUP62	Nucleoporin 62
6	1WWU	EGFR	Epidermal growth factor receptor
7	2PPH	MAPK3	Mitogen-activated protein kinase 3
8	1MYP	MPO	Myeloperoxidase
9	2PFF	FASN	Fatty acid synthase
10	1MJT	NOS2	Nitric oxide synthase 2
11	1GWI	CYP3A4	Cytochrome P450 family 3 subfamily A member 4
12	2ILK	IL10	Interleukin 10
13	3F4M	TNF	Tumor necrosis factor

**Figure 2:**
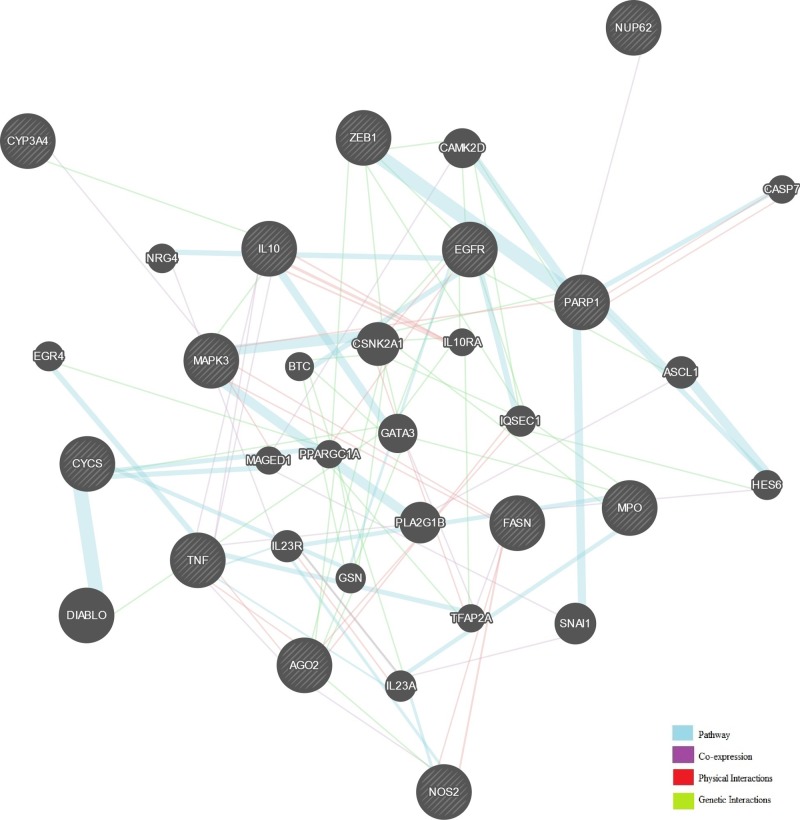
Network of potential pristimerin targets Black protein nodes indicate target proteins, and different connecting colors represent different correlations. Functional association of targets was analyzed using GeneMANIA.

Of 13 targets and their interacting proteins, 58.93% shared the same pathway, whereas 20.78% displayed similar co-expression characteristics. Other results including pathways, physical interactions, and co-localization are presented in Figure 2.

### GO and pathway analysis and network construction

**Figure 3:**
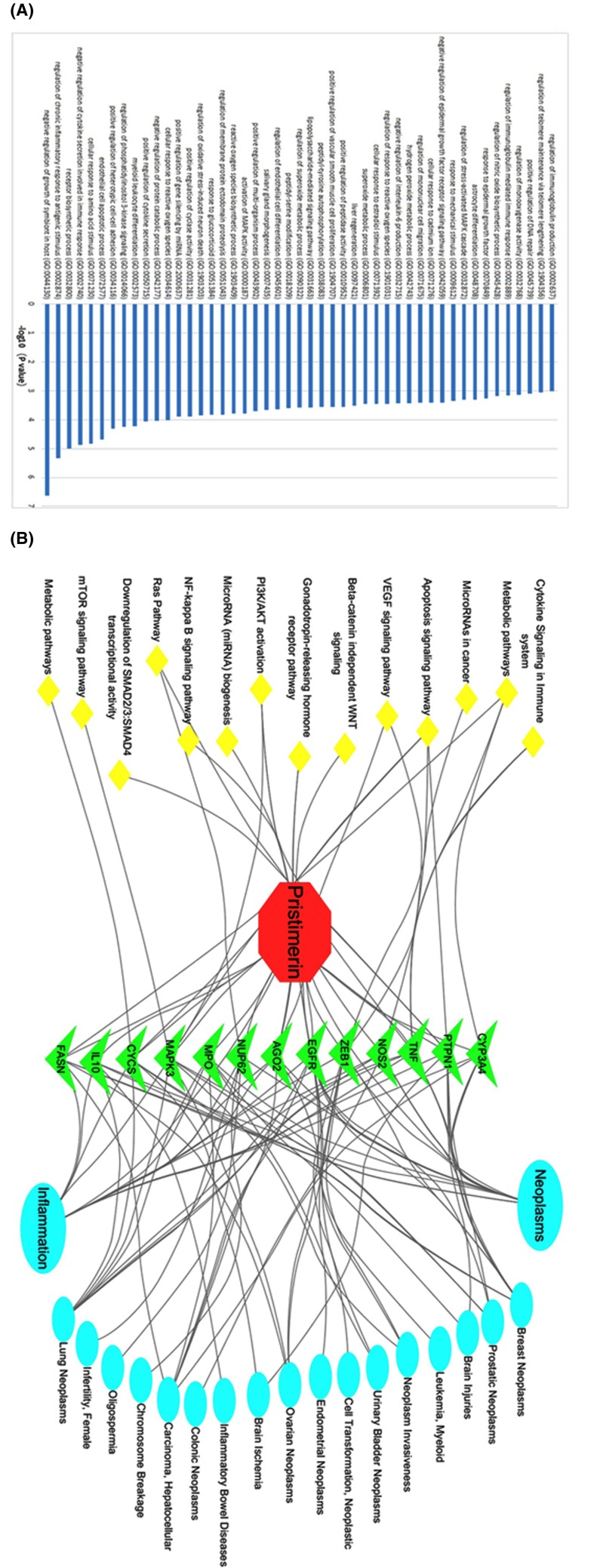
Gene Ontology (GO) analysis of targets and pristimerin-target-pathway network (**A**) Gene Ontology (GO) analysis of targets. The *y*-axis shows significantly enriched biological process categories of the targets, and the *x*-axis shows the enrichment scores of these terms (*P*<0.05). (**B**) Pristimerin-target-pathway network. Red octagon = pristimerin, green triangle = target proteins, blue ellips = disease, yellow diamond = pathway.

Analysis of interaction network regulation was performed using PANTHER to further investigate the 13 identified targets. As shown in Figure 3A, hub genes were significantly enriched in biological processes. In particular, the top six enriched functions were the negative regulation of symbiont growth in the host (GO: 0044130), regulation of chronic inflammatory response to antigenic stimulus (GO: 0002874), receptor biosynthetic process (GO: 0032800), negative regulation of cytokine secretion involved in immune response (GO: 0002740), cellular response to amino acid stimulus (GO: 0071230), and endothelial cell apoptotic process (GO: 0072577).

An entire network was constructed using Cytoscape 3.0 based on target fishing and pathway analysis. As shown in Figure 3B, a hub of genes related to pristimerin was primarily associated with tumor and inflammation, additional clusters associated with miRNA, apoptosis signaling pathway, and cytokine signaling in the immune system were also enriched.

### PTPN1 and AGO2 mutation was correlated with a survival rate in diffuse glioma

**Figure 4:**
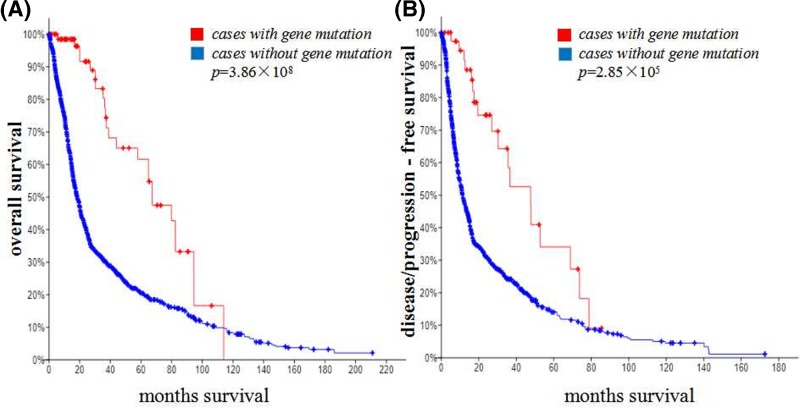
Survival of patients with or without both PTPN1 and AGO2 genes mutation (**A**) Overall survival of patients with or without both PTPN1 and AGO2 genes mutation. (**B**) Disease/progression-free survival of patients with or without both PTPN1 and AGO2 genes mutation.

For the 13 hub genes, a network was analyzed using the cBioPortal online platform. The overall survival analysis of the hub genes was performed using the Kaplan–Meier curve. Defuse glioma patients with PTPN1, and AGO2 mutation showed worse overall survival both in the overall group (*p* = 3.86 × 10^−8^; Figure 4A) and disease-free/progression-free group (*P*=2.85 × 10^−5^; Figure 4B), indicating that PTPN1 and AGO2 may play important roles in diffuse glioma and were related to survival rate.

### Pristimerin inhibited the growth of glioma cells and induced apoptosis in glioma cells

**Figure 5:**
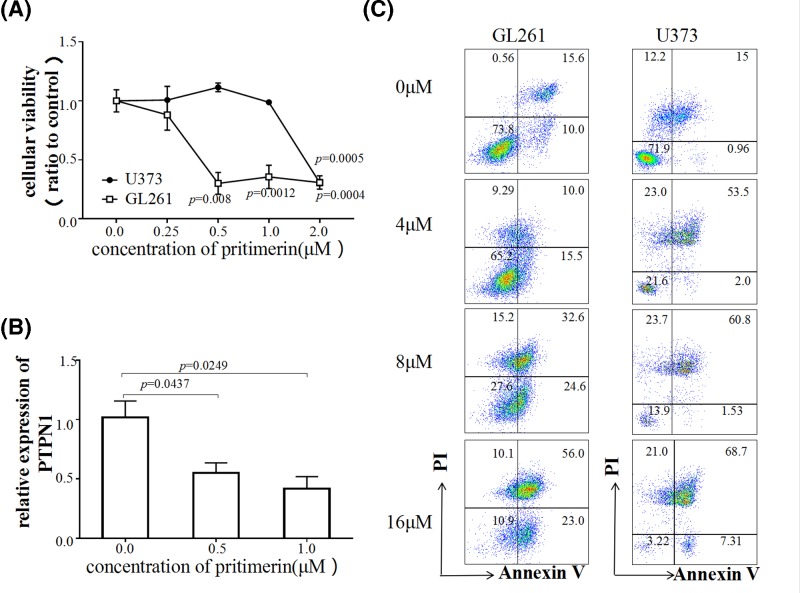
Pristimerin inhibits the cellular viability, and induces apoptosis in glioma cells (**A**) CCK-8 assay showed that pristimerin inhibited the viabilities of U373 and GL261 glioma cells in a dose-dependent manner. (**B**) Pristimerin inhibited the expression of PTPN1 in U373. (**C**) Flow cytometry analysis with Annexin V and PI double staining proved that apoptosis was induced in glioma cells.

To investigate the potential role of PTPN1, cell viability was detected using the CCK-8 assay (with pristimerin concentrations up to 2 μM, *P*=0.0005; Figure 5A). The result showed that pristimerin at a concentration of 0–1 μM did not significantly affect U373 cell viability; however, the proliferation of glioma cells was remarkably inhibited when the concentration was brought up to 2 μM. Meanwhile, GL261 cell viability was severely inhibited when the concentration used in the experiment was 0.25 μM. Subsequently, the expression level of PTPN1 with the higher concentrations was analyzed by qRT-PCR. The results indicated that the PTPN1 level in U373 cells significantly decreased in a dose-dependent manner, starting at 0.5 μM, prior to the change in cell viability (all *P*<0.05; Figure 5B). In addition to the cell growth experiments, to confirm the effect of pristimerin on apoptosis, we elevated the concentration to 4−16 μM and performed a double-staining with Annexin V-FITC/PI and assessed fluorescence by flow cytometry. The results showed that pristimerin increased the Annexin V^+^ population in glioma cells (Figure 5C). Our findings suggest that in the presence of a low-concentration of pristimerin, cell viability and expression of PTPN1 were significantly down-regulated, while pristimerin levels were elevated; and higher concentration of pristimerin could induce apoptosis in glioma cells.

### miR-542-5p targeted AGO2 and PTPN1 in glioma cells

**Figure 6:**
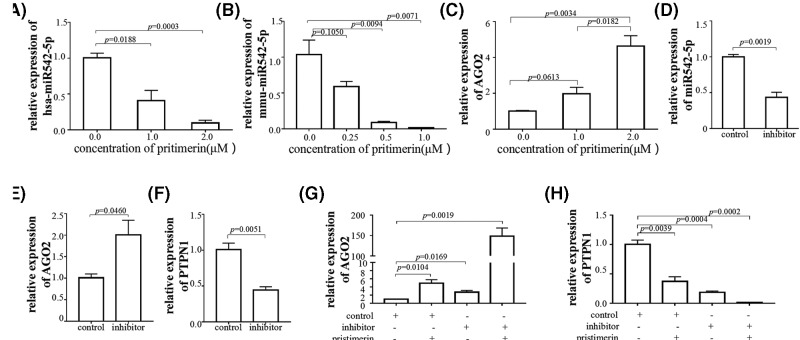
miR-542-5p targeted AGO2 and PTPN1 in glioma cells (**A**) Expression of hsa-miR-542-5p decreased in U373 24 h post-treated with pristimerin, and in a dose-dependent manner. (**B**) Expression of mmu-miR-542-5p decreased in GL261 24 h post-treated with pristimerin, and in a dose-dependent manner. (**C**) Expression of AGO2 elevated in U373 24 h post-treated with different concentration of pristimerin. (**D**) Levels of miR-542-5p 48 h after transfection of miR-542-5p control and inhibitor in U373. (**E**) Levels of AGO2 mRNA elevated 48 h after transfection of miR-542-5p inhibitor than control in U373. (**F**) Levels of PTPN1 mRNA decreased 48 h after transfection of miR-542-5p inhibitor than control in U373. (**G**) Levels of AGO2 mRNA expression 48 h after transfection of miR-542-5p inhibitor or control, and 24 h post-treated with or without pristimerin (1 μM) in U373. (**H**) Levels of PTPN1 mRNA expression 48 h after transfection of miR-542-5p inhibitor or control, and 24 h post-treated with or without pristimerin (1 μM) in U373.

miRNA has been confirmed to guide AGO2 to its specific targets through sequence complementarity, which subsequently located in RISC leads to mRNA cleavage or translation inhibition. The research associated with miR-542 was focused on inhibiting the survival, proliferation, migration, angiogenesis, and metastasis of tumor cells [20,30,31]. Hence, we used different pristimerin concentrations to treat glioma cells. We observed that with elevated pristimerin concentration, miR-542-5p and PTPN1 expression were negatively associated while AGO2 expression was positively correlated. Therefore, it was speculated that miR-542-5p might directly modulate AGO2 into RISC, after which it would be degraded. The RISC subsequently leads to repression of PTPN1, result in the proliferation inhibited by pristimerin. To verify the hypothesis, we used siRNA at 48 h after transfection with miR-542-5p inhibitor; the AGO2 mRNA level was higher while PTPN1 was lower, indicating that miR-542-5p might act upstream of AGO2 and PTPN1. We next detected the effect of pristimerin treatment with or without miR-542-5p silence in glioma cells. The glioma cells were transfection of miR-542-5p inhibitor and control for 48 h, and treated with or without pristimerin (1 μM) for 24 h, then qRT-PCR was performed to determine the expression of AGO2 and PTPN1. The results suggested that miR-542-5p inhibitor transfection with pristimerin treatment enhance expression of AGO2 and decrease expression of PTPN1 significantly, which mean that pristimerin and miR-542-5p silence had the synergistic effect in glioma cells (Figure 6). The above findings revealed that the inhibition effects of miR-542-5p on cell proliferation were potentially achieved by its regulation on AGO2 and PTPN1 expression, which involved in diffuse glioma progression.

## Discussion

Pristimerin is an active ingredient extracted from traditional medicinal plants. Some studies have shown that pristimerin can inhibit tumor angiogenesis and drug resistance development [32,33]. In the present study, two compounds datasets were analyzed to obtain potential target proteins of pristimerin. Of these, 13 targets proteins associated with various pharmacological activities were identified. GO, and pathway analysis were performed to construct a drug target-disease association network. The results indicated that pristimerin has various functions, including anticancer and anti-inflammatory activities. Pristimerin is predicted to target various proteins and pathways to form a network that exerts systemic pharmacological effects.

In the present study, we observed that, with PTPN1 and AGO2 mutation, patients with diffuse glioma had a lower survival rate. Furthermore, we proved that in low concentration, pristimerin inhibited the viability of glioma cells through AGO2, and suppressed expression PTPN1 via a canonical miRNA-mediated silencing mechanism, while in high concentration, pristimerin induced apoptosis. It has already been reported that miR-132 can directly interact with the miR-132 binding site in AGO2 3′-UTR and that miR-132 overexpression suppresses AGO2 expression [34]. This is in agreement with the results of our study in which miR-542-5p might be an upstream regulator of AGO2, contributing to PTPN1 cleavage or translation inhibition. miRNA plays a critical role in biological and cellular processes, which are correlated with the development of organisms and diseases, including malignant transformation. AGO2 is an essential component of the RISC and plays a central role in RNA silencing processes [35]. The RISC is responsible for the gene-silencing phenomenon known as RNA interference. After the treatment with antitumor drugs, the expression levels of a large number of miRNAs could be affected, shaping a specific miRNA expression profile footprints [36]. As a novel pharmaceutical, the miRNAs that are differentially expressed after the treatment with pristimerin may play a key regulatory role in the proliferation and apoptosis of glioma cells, and slow the disease progression.

Previous studies have reported that miR-542 was a multifunctional miRNA, which is involved in cell apoptosis, proliferation, invasion, and other biological processes [20,30,31]. Two mature sequences of miR-542-5p and miR-542-3p were formed from pre-miR-542. Of note, most of the current studies focused on the effects of miR-542-3p on tumor cells. In general, miR-542-3p is a known tumor suppressor miRNA, and the overexpression of miR-542-3p can inhibit the survival, proliferation, migration, angiogenesis, and metastatic potential of tumor cells [37–39]. While studies on miR-542-3p abound, there are only a few studies on miR542-5p, and its role is still controversial. In the present study, we first demonstrated that the expression of miR-542-5p was significantly lower with elevated pristimerin concentration accompany with glioma cell viability declined, which indicated that miR-542-5p might facilitate glioma cell proliferation. Subsequently, the effects of miR-542-5p on RISC and proliferation associated gene AGO2 and PTPN1 were evaluated in glioma cells. Results indicated that post-treatment with pristimerin and miR-542-5p silence had profoundly the same effect, elevated AGO2 and decreased PTPN1, which might be related with cell proliferation, lead to glioma progression.

As we predicted, several pathways were related to the biochemical activity of pristimerin, and apoptosis progression could me mediated by various mechanisms, such as caspase activation [40,41], changes in proteasome [42], and inhibition of anti-apoptotic factors, including nuclear factor-κB (NFκB) [43], Akt [44], and mammalian target of rapamycin (mTOR) [45]. Moreover, PTPN1 is a gene associated with NFκB pathway [46], which encoded PTP1B. When activated, the inhibitor of κB (IκB) kinase phosphorylates IκB regulatory domain, leading to its degradation, which releases the p65 subunit of NFκB leading to the translocation of NFκB to the nucleus and subsequent transcription off PTP1B, suggesting that the activation of NFκB pathway mediates expression of PTP1B. Our findings provided the basis for further exploring the application of pristimerin as a novel anticancer treatment. However, further studies are needed.

## Conclusions

In summary, the present study revealed that pristimerin participated in the proliferation, apoptosis of glioma cells and demonstrated that AGO2 and PTPN1 were partially responsible for the miR-542-5p induced inhibition of cell proliferation.

## Abbreviations

ADME: absorption, distribution, metabolism, and excretion
AGO: Argonaute
DL: drug-likeness
DMEM: Dulbecco’s modified Eagle’s medium
GBM: glioblastoma multiform
NFκB: nuclear factor-κB
OB: oral bioavailability
PTP1B: protein tyrosine phosphatase 1B
PTPN1: protein tyrosine phosphatase, non-receptor type 1
RISC: RNA-induced silencing complex
